# A Hybrid Model of Maximum Margin Clustering Method and Support Vector Regression for Noninvasive Electrocardiographic Imaging

**DOI:** 10.1155/2012/436281

**Published:** 2012-11-01

**Authors:** Mingfeng Jiang, Feng Liu, Yaming Wang, Guofa Shou, Wenqing Huang, Huaxiong Zhang

**Affiliations:** ^1^School of Information Science and Technology, Zhejiang Sci-Tech University, Hangzhou 310018, China; ^2^School of Information Technology and Electrical Engineering, University of Queensland, St. Lucia, Brisbane, QLD 4072, Australia; ^3^School of Electrical and Computer Engineering, University of Oklahoma, Norman, OK 73019, USA

## Abstract

Noninvasive electrocardiographic imaging, such as the reconstruction of myocardial transmembrane potentials (TMPs) distribution, can provide more detailed and complicated electrophysiological information than the body surface potentials (BSPs). However, the noninvasive reconstruction of the TMPs from BSPs is a typical inverse problem. In this study, this inverse ECG problem is treated as a regression problem with multi-inputs (BSPs) and multioutputs (TMPs), which will be solved by the Maximum Margin Clustering- (MMC-) Support Vector Regression (SVR) method. First, the MMC approach is adopted to cluster the training samples (a series of time instant BSPs), and the individual SVR model for each cluster is then constructed. For each testing sample, we find its matched cluster and then use the corresponding SVR model to reconstruct the TMPs. Using testing samples, it is found that the reconstructed TMPs results with the MMC-SVR method are more accurate than those of the single SVR method. In addition to the improved accuracy in solving the inverse ECG problem, the MMC-SVR method divides the training samples into clusters of small sample sizes, which can enhance the computation efficiency of training the SVR model.

## 1. Introduction 

The technique of noninvasive imaging of the heart's electrical activity from the body surface potentials (BSPs) constitutes one form of the inverse problem of ECG [1, 2]. Approaches to solving the inverse ECG problem have been usually based on either an activation-based model or a potential-based model, which includes epicardial, endocardial, or transmembrane potentials. Activation-based models are used to investigate the arrival time of the propagation wavefront within the myocardium [3, 4]. The potential-based models are used to evaluate the potential values on the cardiac surface [5–7] or within the myocardium [8] at certain time instants. In this study, we explore a new solution for ECG inverse problem using the potential-based approach.

Due to its inherent ill-posed property, the inverse ECG problem is usually solved by “regularization” techniques. In the last decades, numerous regularization methods have been proposed to solve this ill-posed problem, including truncated total least squares (TTLS) [9], GMRes [10], and the LSQR [11, 12]. Most of them are essentially L2-norm based regularization schemes, which inherently lead to considerable smoothness of the inverse solutions. L1-norm regularization method can overcome this drawback of L2-norm regularization method, which has been applied for epicardial potential reconstruction [13–15]. Although the above-mentioned regularization methods can more or less deal with the geometry and measurement noises for the ECG inverse problems, which depends on the regularization parameters, the robustness of the inverse solution is not always guaranteed. In this paper, without seeking assistance from the regularization techniques, we explore an alternative, more robust approach to solve the inverse ECG problem. The method is called Support Vector Regression (SVR) [16]. To find the solution for the inverse ECG problem, a regression model will be set up with multi-inputs (BSPs) and multioutputs (transmembrane potentials, TMPs). This statistic method based solution will be assessed with the quality of the inversely predicated TMPs from the measured BSPs. Compared with conventional regularization methods (e.g., zero order Tikhonov and LSQR), the SVR method can produce more accurate results in terms of reconstruction of the transmembrane potential distributions on epi- and endocardial surface. In addition, when the PCA and KPCA are adopted to extract useful features from the original inputs for building the SVR model, the SVR method with feature extraction (PCA-SVR and KPCA-SVR) outperforms that without the extract feature extraction (single SVR) in terms of the reconstruction of the TMPs [17]. 

Compared with using single SVR model, the hybrid models by integrating difference methods show better performance. The self-organizing map (SOM) is an unsupervised and competitive learning algorithm, which can be viewed as clustering techniques [18]. Combining the SOM with SVR or LS-SVM, the proposed hybrid method has the potential to find better inverse solutions than using a single SVR model [19, 20]. Xu et al. [21] proposed the Maximum Margin Clustering (MMC) method, which performs clustering by simultaneously finding the large margin separating hyperplane between clusters. The MMC method has been successfully applied to many clustering problems [22]. However, its efficiency is an issue of concern. Recently, Zhang et al. proposed [23, 24] an efficient approach for solving the MMC via an alternative optimization procedure, which was implemented by using the SVR method with the Laplacian loss in the inner optimization subproblem. The modified MMC algorithm is more accurate, much faster and therefore more practical for solving engineering inverse problems. In this paper, the hybrid model of modified MMC method and SVR is proposed to solve the inverse ECG problem, which is referred to as an MCC-SVR method. The conference version of this submission has appeared in CINC 2011 [25]. This submission has undergone substantial revisions and offers extended experiment results.

The main purpose of this study is to use an MCC-SVR model to investigate the reconstruction capability of TMPs. In this study, based on our previously developed realistic heart-torso model, the equivalent double layer (EDL) source model method was applied to generate the data set for training and testing the SVR model. The proposed algorithm was also compared with a single SVR model for noninvasive ECG imaging.

## 2. Theory and Methodology

**Figure 1:**
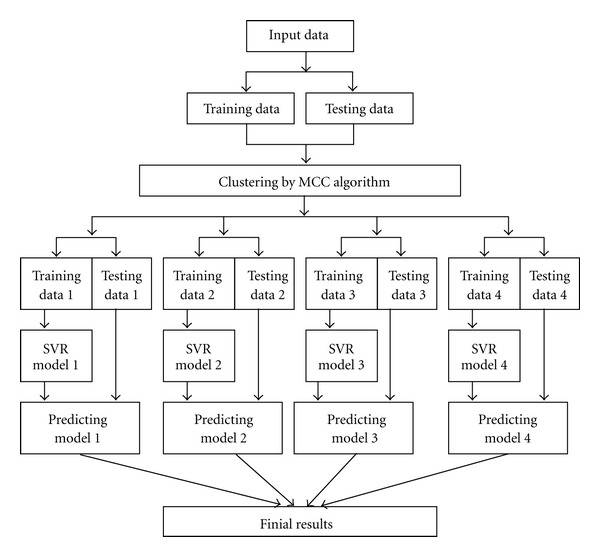
The framework of the proposed MCC-SVR method.

The framework of the proposed MCC-SVR method is shown in Figure 1. The MCC method is used to classify the input data; the SVR is then applied to construct the regression model of each cluster. 

### 2.1. Maximum Margin Clustering (MMC) Method [23, 24]

The clustering principle is to find a labeling to identify dominant structures in the data and to group similar instances together, so the margin obtained would be maximal over all possible labelings, that is, given a training set {(*x*_*i*_,*y*_*i*_)}_*i*=1_^*n*^, where *x*_*i*_ ∈ *χ* is the input and *y*_*i*_ ∈ {±1} is the output. The SVM finds a large margin hyperplane to separate patterns of opposite classes by the classify function *f*(*x*) [26]:

(1) $$\begin{array}{c} f\left(x\right)=\omega^{T}\varphi \left(x\right)+b, \end{array}$$

where *φ*(*x*) denotes the high-dimensional feature space, which is nonlinearly mapped from the input space *x* by the kernel function *k*, *ω* is the normal vector of the hyperplane, and *b* is the offset of the hyperplane. Computationally, this leads to the following optimization problem [24, 26]:

(2) min⁡ω,b,ξ ||ω||2+2CξTesubject  to {yi(ωφ(xi)+b)≥1−ξiξi≥0, i=1,…,n,

where *ξ* = [*ξ*_1_,…, *ξ*_*n*_]^*T*^ is the vector of a slack variable for the errors, and *C* > 0 is the trade-off parameter between the smoothness ||*ω*||^2^ and the fitness (*ξ* ^*T*^ *e*) of the decision function *f*(*x*).

MMC attempts to extend large margin methods to allocate the input data points to different classes, leading to large separation between the different classes. Here, the case with two clusters is considered in this work. Since one could simply assign all the data points to the same class and obtain an unbounded margin, a proper constraint on the class balance needs to be imposed. Xu et al. [21] introduced a class constraint that requires *y* to satisfy

(3) $$\begin{array}{c} -\ell \leq e^{T}y\leq \ell , \end{array}$$

where *ℓ* ≥ 0 is a user-defined constant controlling the class imbalance. Then the margin is maximized with respect to both unknown *y* and unknown SVM parameter (*ω*, *b*) as follows:

(4) min⁡ ymin⁡ω,b,ξ⁡ ||ω||2+2CξTesubject  to {yi(ωφ(xi)+b)≥1−ξiξi≥0, yi∈{±1}, i=1,…,n−ℓ≤eTy≤ℓ.

The origin nonconvex MMC problem in (4) can be formulated as a sequence of QPs which can be solved by some efficient QP solvers. However, it suffers from a premature convergence and easily gets stuck in poor local optima. Zhang et al. [23, 24] proposed to replace the SVM by SVR with Laplacian loss, which can lead to a significant improvement in the clustering performance compared to that of iterative SVM procedure. The primal problem of SVR with Laplacian loss can be formulated as

(5) min⁡ω,b,ξi,ξi∗⁡ ||ω||2+2C∑i=1n(ξi+ξi∗)subject  to {yi−(ωTφ(xi)+b)≤ξi(ωTφ(xi)+b)−yi≤ξi∗ for  i=1,…,n,ξi≥0,  ξi∗≥0,

where *ξ*_*i*_ and *ξ*_i_* are slack variables. With the obtained labels, the MMC problem based on the iterative SVR with the Laplacian loss becomes

(6) min⁡ω,b,ξi,ξi∗⁡ ||ω||2+2C∑i=1n(ξi+ξi∗)subject  to {yi−(ωTφ(xi)+b)≤ξi(ωTφ(xi)+b)−yi≤ξi∗ξi≥0, ξi∗≥0 for  i=1,…,nyi∈{±1}−ℓ≤eTy≤ℓ.

After *ω* is obtained from the optimization of SVR, the problem in (6) is reduced to the form

(7) min⁡y,b⁡ ∑i=1n|(ωTφ(xi)+b)−yi|subject  to {yi∈{±1}, i=1,…,n−ℓ≤eTy≤ℓ.

According to Zhang's proposition [24], for a fixed *b*, the optimal strategy to determine the *y*_*i*_'s in (7) is to assign all *y*_*i*_'s as −1 for those with *ω* ^*T*^ *φ*(*x*_*i*_) + *b* < 0 and assign *y*_*i*_'s as 1 for those with *ω* ^*T*^ *φ*(*x*_*i*_) + *b* > 0. The bias *b* can be determined as follows. (i) we sort the *ω* ^*T*^ *φ*(*x*_*i*_)'s and use the set of midpoints between any two consecutive sorted values as the candidates of *b*; (ii) from these sorted *b*'s, the first and the last (*n* − *ℓ*)/2 of them can be dropped, and the middle *ℓ* can be remained; (iii) for each remaining candidate, we determine the *y*_*i*_'s according to the above proposition and compute the corresponding objective value in (7); (iv) finally, we choose the *b* that has the smallest objective. The complete iterative SVR procedure for MCC method is shown in Algorithm 1.

### 2.2. Support Vector Regression (SVR) Model

The SVR algorithm [26] is only briefly described here; for details, see [16, 26]. As a linear regression model, the SVR algorithm relies on an estimation of a linear regression function:

(8) $$\begin{array}{c} f\left(x\right)=\langle \omega ,x\rangle +b,\;\left(\omega ,x\in ℜ\right), \end{array}$$

where *ω* and *b* are the slope and offset of the regression linear, and 〈·, ·〉 denotes the dot product in *ℜ*. The above regression problem can be written as a convex optimization problem:

(9) min⁡ 12||ω||2subject  to {yi−〈ω,xi〉−b≤ε〈ω,xi〉+b−yi≤ε.

In (9), an implicit assumption is that a function *f* essentially approximates all pairs (*x*_*i*_, *y*_*i*_) with *ε* precision, but sometimes this may not be the case. Therefore, one can introduce two additional positive slack variables *ξ*_*i*_, *ξ*_i_* to refine the estimation of variables *ω* and *b*. Now (9) can be reformulated [16] as

(10) min⁡ 12||ω||2+C∑i=1n(ξi+ξi∗)subject  to {yi−〈ω,xi〉−b≤ε+ξi〈ω,xi〉+b−yi≤ε+ξi∗ξi,ξi∗≥0,

where the constant *C* is a trade-off parameter and *n* denotes the number of samples; *ξ*_*i*_ represents the upper training error, and *ξ*_i_* is the lower training error subject to *ε* intensive tube. According to the strategy outlined by Vapnik [26], using Lagrange multipliers, the constrained optimization problem shown in (3) can be further restated as the following equation:

(11) f(x,αi,αi∗)=∑i=1n(αi−αi∗)K(xi,x)+bsubject  to ∑i=1n(αi−αi∗)=0, 0≤αi,  αi∗≤C,

where *α*_*i*_ and *α*_i_* are the Lagrange multipliers. The term *K*(*x*_*i*_, *x*_*j*_) in (11) is defined as the kernel function, whose values are the inner product of two vectors *x*_*i*_ and *x*_*j*_ in the feature space *φ*(*x*_*i*_) and *φ*(*x*_*j*_). And bias *b* can be computed as follows:

(12) $$\begin{array}{c} b=\{\begin{array}{c} y_{i}-\sum_{j=1}^{n}\left(\alpha_{i}-\alpha_{i}^{*}\right)K\left(x_{j},x_{i}\right)-\varepsilon \;\text{for}\;\;\alpha_{i}\in \left(0,C\right)\\ y_{i}-\sum_{j=1}^{n}\left(\alpha_{i}-\alpha_{i}^{*}\right)K\left(x_{j},x_{i}\right)+\varepsilon \;\text{for}\;\;\alpha_{i}^{*}\in \left(0,C\right). \end{array} \end{array}$$

The kernel function handles any dimension feature space with no explicit calculation of *φ*(*x*). In this study, the Gaussian kernel function is chosen as the SVR's application mapping in this study:

(13) $$\begin{array}{c} K\left(x_{i},x_{j}\right)=\exp\left(-\frac{{||x_{i}-x_{j}||}^{2}}{2\sigma^{2}}\right), \end{array}$$

where *x*_*i*_ and *x*_*j*_ are input vector spaces; *σ* ^2^ is the bandwidth of the kernel function.

**Figure 2:**
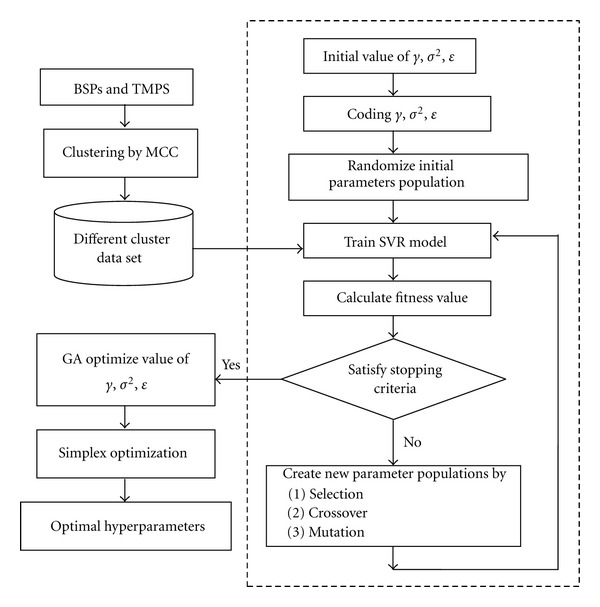
GA-Simplex optimization procedure for the parameter selection in the MCC-SVR model.

In this study, an accurate and fast approach based on the GA and the simplex search techniques is presented to determine the optimal hyperparameters of the SVR model [17], as shown in Figure 2. The GA algorithm used here is based on a GA toolbox developed by Chipperfield et al. [27], and the simplex optimization method is implemented using the MATLAB optimization toolbox. The developed SVR model was trained and validated with the software LIBSVM [28].

### 2.3. Simulation Protocol and Data Set

**Figure 3:**
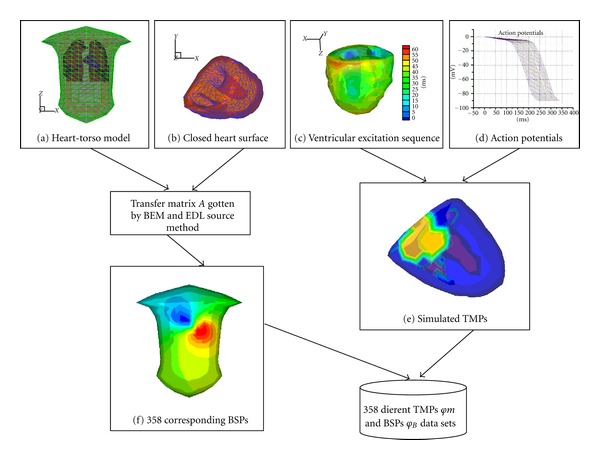
The block diagram of the simulation protocol for the construction of the data sets.

The SVR model is tested with our previously developed realistic heart-torso model [6, 17]. In this study, an equivalent double layer (EDL) source model is adopted to simulate the cardiac equivalent source, which represents the cardiac electrical activity by means of double layer source on the closed surface (including the endo- and epicardial surface of ventricle). For the ECG inverse problem studies, the ventricular surface TMPs and body surface potentials (BSPs) are evaluated based on the EDL source model. The transfer matrix *A* between TMPs and BSPs is evaluated by the boundary element method (BEM), and it has the dimension of 412 × 478 and its condition number (the ratio of largest and smallest singular values) is 5.6 × 10^12^. As shown in Figure 3, the EDL source method is used to obtain the BSPs *φ*_*B*_ and the TMPs *φm*. For the construction of the training and testing data set Different Action Potentials (APs) for various myocardial cells and the normal Ventricular Excitation Sequence (VES) are used to calculate the TMPs (*φm*) at different times; from the calculated TMPs, the corresponding BSPs are deduced with the transfer matrix *A*.

In this study, a normal ventricular excitation data set is prepared for the setup of the SVR model. The considered ventricular excitation period from the first breakthrough to the end is 357 ms and the time step is 1 ms, and, thus, 358 BSPs *φ*_*B*_ and TMPs *φm* temporal data sets are numerically recorded; in addition, the 30 dB simulated Gaussian white noise is added into the BSPs *φ*_*B*_ representing the measurement noises. 60 datasets at times of 3 ms, 9 ms, 15 ms,…, and 357 ms after the first ventricular breakthrough are used as testing samples to evaluate the generalization capacity of the proposed SVR model. The rest 298 in 358 data sets are employed as the training samples for building the SVR model. With the consideration of a wide numerical range of the *φ*_*B*_ values, for each time, the *φ*_*B*_ values can be scaled to the range (0, 1):

(14) $$\begin{array}{c} \varphi_{BtN}=\frac{\varphi_{Bt}-\varphi_{Bt\min}}{\varphi_{Bt\max}-\varphi_{Bt\min}}, \end{array}$$

where *φ*_*Bt*_ are the body surface potentials at time instant *t*, *φ*_*Bt*max⁡_ is the maximum value of BSPs at the time *t*, and *φ*_*Bt*min⁡_ is the minimum value of BSPs at the time *t*. 

As the TMPs are known in advance in the simulation study, the accuracy of reconstructed TMPs at the testing time *t* can be evaluated by either relative errors (REs):

(15) $$\begin{array}{c} \text{RE}=\frac{||\varphi_{t}^{c}-\varphi_{t}^{e}||}{||\varphi_{t}^{e}||}, \end{array}$$

or the correlation coefficient (CC), given by

(16) $$\begin{array}{c} \text{CC}=\frac{\sum_{i=1}^{n}\left[{\left(\varphi_{t}^{c}\right)}_{i}-{\overset{-}{\varphi }}_{t}^{c}\right]\left[{\left(\varphi_{t}^{e}\right)}_{i}-{\overset{-}{\varphi }}_{t}^{e}\right]}{||\varphi_{t}^{c}-{\overset{-}{\varphi }}_{t}^{c}||||\varphi_{t}^{e}-{\overset{-}{\varphi }}_{t}^{e}||}, \end{array}$$

where *n* is the number of nodes on the ventricular surface. *φ*_*t*_^*e*^ denotes the simulated TMPs distribution at time *t*, and *φ*_*t*_^*c*^ are inversely computed. The quantities ${\overset{-}{\varphi }}_{t}^{c}$ and ${\overset{-}{\varphi }}_{t}^{e}$ are the mean value of *φ*_*t*_^*c*^ and *φ*_*t*_^*e*^ over the whole ventricular surface nodes at time *t*. 

## 3. Results

**Figure 4:**
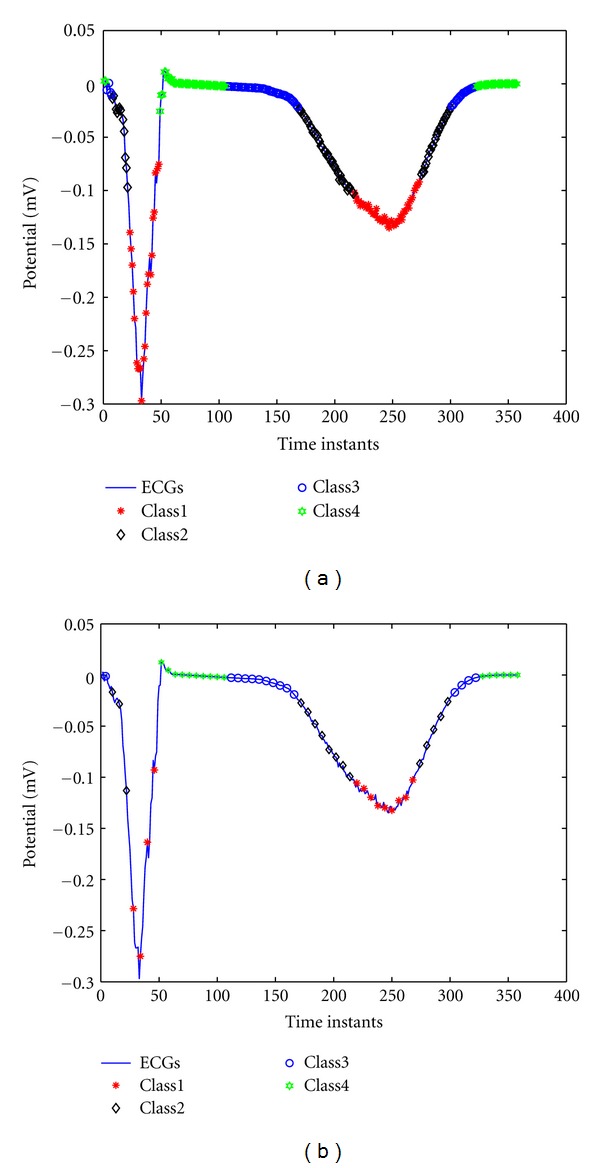
On one representative epicardial point, (a) the 298 training samples are classified into four clusters by using MCC method; (b) for the 60 testing samples, the MCC method is used to find their corresponding clusters.

According to the MCC method, the above 298 training samples are classified four clusters as shown in Figure 4(a), and the numbers of the four clusters is 80, 74, 70, and 74, respectively. Then the individual SVR model is trained for each cluster, and the hyperparameters are determined using the GA-Simplex method. For 60 testing samples, the MCC method is used to find their corresponding clusters, as shown in Figure 4(b). 

**Figure 5:**
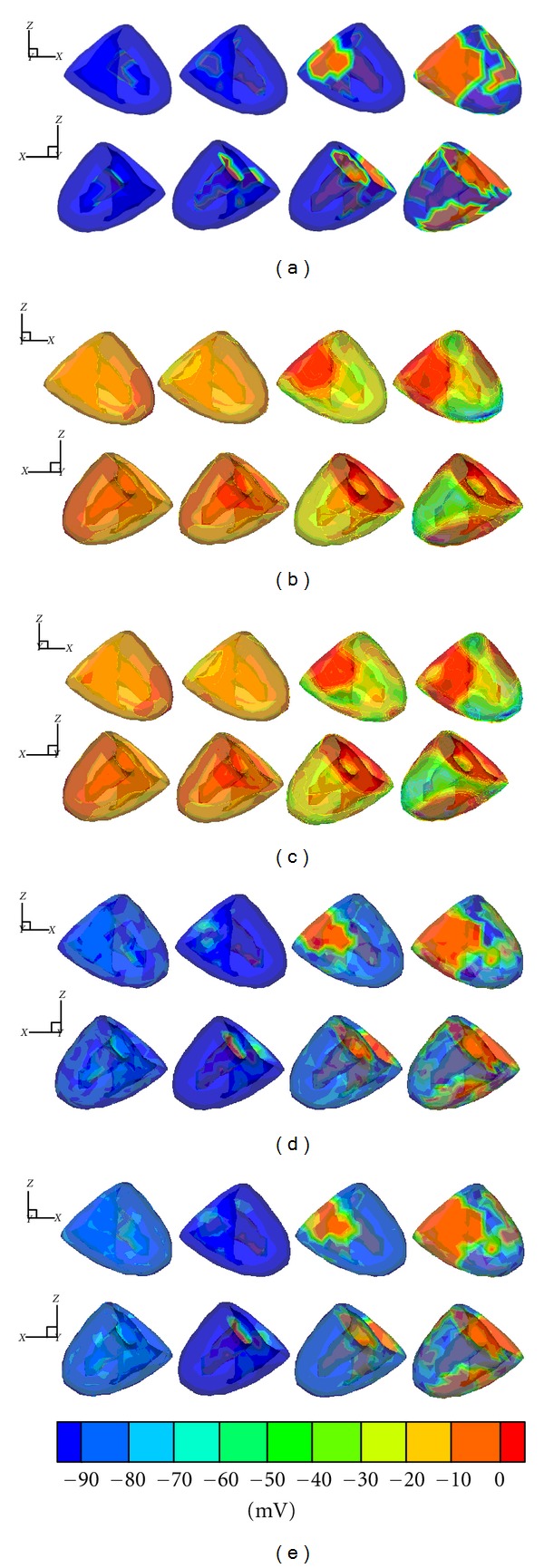
The TMPs distribution on the ventricular surface at four sequential testing time points (3, 15, 27, and 39 ms, respectively, after the first ventricular breakthrough). In each subfigure, the upper row shows the TMPs distribution from an anterior view and the lower from a posterior view. (a) The simulated TMPs by using the equivalent double layer (EDL) source model; (b) the reconstructed TMPs by using zero order Tikhonov method; (c) the reconstructed TMPs by using the LSQR method; (d) the reconstructed TMPS by using the single SVR method; (e) the reconstructed TMPs by using the MCC-SVR method.

**Figure 6:**
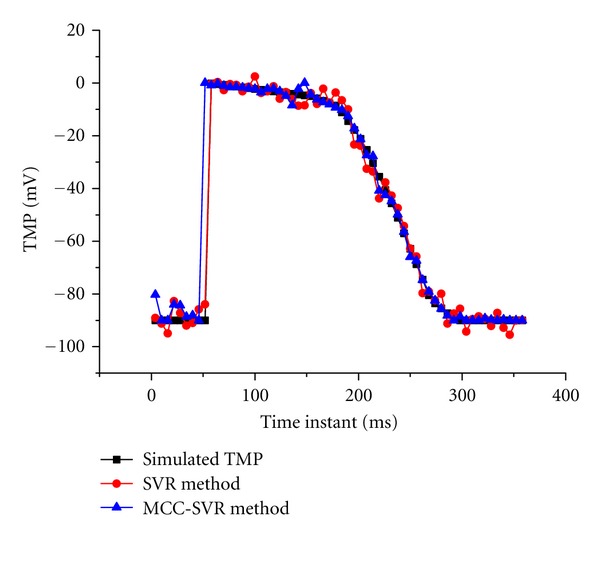
The time courses of the TMPs for one representative source point on the heart surface. The reconstruction TMPs over the 60 testing times with SVR method and the MCC-SVR method are all compared with those simulated TMPs.

To illustrate the performances of the reconstructed TMPs, four sequential testing time points (3, 15, 27, and 39 ms after ventricle excitation) are presented. The inverse ECG solutions are shown in Figure 5; in contrast to the conventional regularization methods, such as zero order Tikhonov regularization method and LSQR regularization method, the single SVR method can yield rather better results with lower RE and higher CC. Moreover, it can be seen that the MCC-SVR method offers superior performances than the single SVR method, as its solution is more close to the simulated TMPs distributions. The time courses of the simulated TMPs and reconstructions for one representative source point on the heart surface are depicted in Figure 6. It can be found that, in reconstructing the TMPs for one representative source point over all the testing times, the MCC-SVR method offers better solution compared with single SVR method.

**Figure 7:**
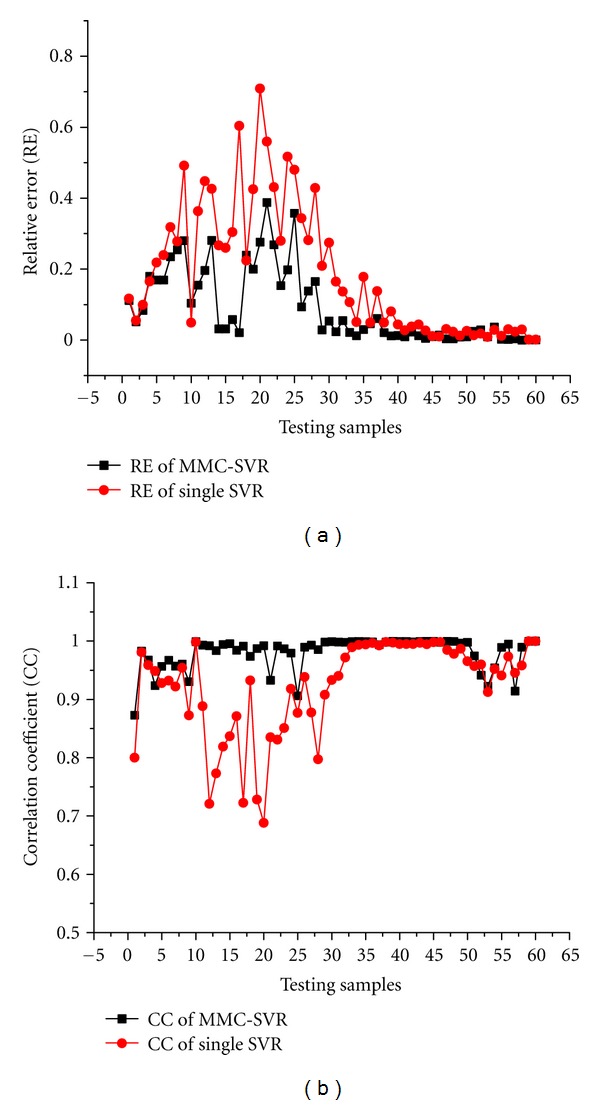
The performances of the reconstructed TMPs over 60 sampling times by using SVR and MCC-SVR, respectively. (a) The REs of the reconstructed TMPs; (b) the CCs of the reconstructed TMPs.

The RE and CC of the reconstructed TMPs with different methods can be found in Figure 7. In contrast to the single SVR method, the MCC-SVR method can yield improved results with a lower RE and a higher CC over the 60 testing samples. 

By dividing the training samples into smaller clusters, the training time for each cluster can be reduced. The training times for each cluster by using the MCC-SVR method are 6715.9 seconds, 6821.1 seconds, 4550.6 seconds, and 5162.4 seconds, respectively, and the total time of the four clusters is 23250 seconds. When using the single SVR model to train the model of the all training samples, it takes 35233.4 seconds.

## 4. Discussion and Conclusion

In this study, MCC-SVR method is proposed to solve the noninvasive ECG imaging problem. Here, the MMC approach is adopted to cluster the training samples firstly, and then SVR method is applied to construct the model for each cluster. After building different cluster models, for the testing sample, we can find its matched cluster and then use the corresponding SVR model to reconstruct the TMPs. From the reconstructed TMPs as shown in Figures 5 and 6, it can be seen that the MCC-SVR methods offer better solution compared with single SVR method. According to the evaluation indices RE and CC, the performances of the reconstructed TMPs by using the MCC-SVR can constantly converge to a smaller RE and a higher CC on the testing samples than those of the single SVR method, as shown in Figure 7. In terms of the computation efficiency of the training SVR model, for the given training samples, the MCC-SVR method can save about 34% time than the single SVR method. With the increasing of the training samplings, the MCC-SVR method lessens more training time than the single SVR method. Moreover, the training process for each cluster can be implemented simultaneously using parallel computing, therefore further enhance the training efficiency.

In summary, this paper proposed the MCC-SVR method for the inverse solutions of the ECG problem. The new algorithm was tested and compared with single SVR schemes using a realistic heart-torso model. The experimental results show that the MCC-SVR can improve the generalization performance of the single SVR in reconstructing the TMPs, leading to a more accurate reconstruction of the TMPs. In our future work, we plan to improve the MCC-SVR method for solving various nonlinear regression problems in noninvasive ECG imaging.

## Figures and Tables

**Algorithm 1:**
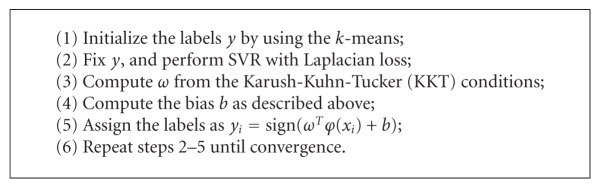
Iterative SVR procedure for MCC method.
